# Editorial: Protective Resources for Psychological Well-Being of Adolescents

**DOI:** 10.3389/fpsyg.2020.00720

**Published:** 2020-04-15

**Authors:** Lourdes Rey, Mario Pena, Félix Neto

**Affiliations:** ^1^Department of Psychology, Evaluation and Psychological Treatment, University of Malaga, Málaga, Spain; ^2^Department of Methods of Research and Diagnosis in Education II, National University of Distance Education, Madrid, Spain; ^3^Department of Psychology, University of Porto, Porto, Portugal

**Keywords:** protective factors, adolescence, well-being, education, mental-health

Adolescence is an essential stage of the lifecycle, in which children begin to develop their identity, experiment with multiple changes in several areas and contexts (family, school, social, and personal) and cope with stressful events. How they manage each changing situation will influence their psychological adjustment and personal well-being. Positive Psychology constitutes a framework focusing on positive aspects, personal resources and protective factors that prevent, provide a buffer against and boost coping with stressful and difficult situations. Therefore, the main aim of this Research Topic is to examine the role of protective factors in family, school, social relationships and personal contexts in preventing psychological maladjustment and enhancing adolescents' psychological well-being.

For adolescents there are several important contexts that can influence how vulnerable or resilient they are in stressful situations. One of these contexts, considered to be one of the most important and immediate in which adolescents satisfy their basic needs, grow, learn and develop, is that of the family. Thus, Zhao et al. examined the regulatory effect of parental control on the association between sensation-seeking and tobacco and alcohol use among Chinese adolescents. They found that sensation-seeking predicted the use of tobacco and alcohol. However, their findings also revealed that parental psychological control enhanced and moderated the relationship between adolescents' sensation-seeking and their use of tobacco and alcohol. On the basis of these results, the authors proposed several ways of preventing or reducing adolescents' use of tobacco and alcohol. In another study of the family context, Liu et al. examined whether self-control moderated the relationship between parent–adolescent relationships and risk-taking behaviors in a sample of Chinese adolescents. Their findings showed that associations between parent–adolescent relationships were indeed moderated by self-control. Therefore, the authors suggested, interventions to reduce adolescent risk-taking should incorporate the personal features and interactions of families.

The second important context in adolescence is that of school. It is in educational centers that adolescents spend a great amount of time, acquire new knowledge and develop their ability to establish relationships with peers and teachers and to cope with stressful situations (emotions, conflict or academic performance, among others). In one study in this area, Carmona-Halty et al. carried out a longitudinal study to examine the mediating role of academic psychological capital (PsyCap) (including hope, efficacy, resilience, and optimism) on the relationship between the satisfaction of basic psychological needs (BPN) and academic performance in a Chilean sample. They found that adolescents with more satisfied BPN at school accumulated more academic PsyCap, implying better academic performance. These findings suggest that increasing the hope, efficacy, resilience, and optimism of students can enhance the school environment. In another study, Lázaro-Visa et al. looked at the influence of several personal competencies and the school environment, together with various socio-demographic characteristics, on life satisfaction in Spanish pre-adolescent and adolescent samples who had suffered bullying. Their findings showed the school environment, self-esteem and emotional repair to predict these adolescents' life satisfaction. Consequently, the authors pointed to the importance of considering both individual and contextual aspects in attempts to improve the life satisfaction of bullied adolescents.

Friends and social relationships are the third relevant context during adolescence. As the adolescents' peer group, they are a critical influence on adolescents' psychological development. Examining this perspective, Del Rey et al. looked at the impact of sexting and the influence of the need for popularity on this phenomenon among Spanish adolescents. Their results showed that greater need for popularity was accompanied by a higher likelihood of adolescents sharing images of themselves. Furthermore, although sexting implied an active emotional impact on the adolescents involved, a short-term negative impact was not apparent. The researchers stressed the relevance of this for future intervention and prevention programmes targeting sexting. Elsewhere, Gómez-López et al. analyzed the stability of psychological well-being (PWB) over time and the influence of romantic relationships on psychological well-being (including self-acceptance, positive interpersonal relationships, autonomy, and life development) in a prospective study of a Spanish sample. Their findings showed medium to high levels of PWB. In addition, romantic relationships were an important predictor of PWB, being related positively to positive interpersonal relationships and life development, and negatively to self-acceptance and autonomy.

Finally, the personal resources and strategies used by adolescents to manage various situations in this stage of their lifecycle will have an effect on their mental health and psychological adjustment. In this area of research, the role of certain strengths and strategies in terms of their consequences on adolescents' positive development and well-being have been analyzed. First, the role of emotional intelligence (EI), together with social support, life satisfaction and depression, in Moroccan students was examined by Lopez-Zafra et al. Their findings revealed EI to be a protective factor against depression, through social support and life satisfaction. Furthermore, EI moderated the relationship between social support and life satisfaction. Likewise, Chen investigated how school type (boarding schools and day schools) influenced the relationship between emotional intelligence (EI), perceived social support and resilience among Chinese adolescents. The results showed that the greater the perceived support from friends, the more positive the relationship between trait EI and resilience. In addition, for adolescents with lower perceived support from friends, the boarding school experience was a better choice for those with high trait EI. The author went on to outline some implications of this study for parents as well as for mental health professionals.

Second, Tang et al. and Suárez et al. pointed to the important role of, respectively, character strengths and the strategies used by adolescents. Tang et al. considered the use of strengths as a mediator for understanding how character strengths were related to adolescents' academic achievement and well-being. In particular, they examined the following character strengths: caring, inquisitiveness, and self-control. Their findings revealed that the use of such strengths could be an explicative mechanism between character strengths and academic achievement. The authors therefore concluded that one practical implication of their findings is to encourage students to use their strengths in this way. In addition, Suárez et al. studied the use of self-motivation strategies involving classmates (i.e., the raising up of others, annulation of others, deception, and comparison) and explored the relation of these with academic goals (including tasks, ego self-enhancing, ego self-defeating, and work avoidance goals) and belief in control and self-efficacy in learning, in two Spanish samples. Their results showed that adolescents who reported lower use of self-motivation strategies presented lower levels of belief in self-efficacy to learn and perform.

Third, Barcaccia et al. tested a model incorporating forgiveness, anger, hedonic balance, and depression in Italian adolescents using a structural equation modeling approach. Their results suggested that forgiveness protects against depression, helping to control and manage anger. On the basis of their findings, the authors outlined various implications for the well-being of adolescents.

Fourth, Fiorilli et al. analyzed the role of self-esteem and interpersonal stressors as predictors of depression manifestations (i.e., depressed mood, sense of inadequacy, and insecurity) among Italian pre-adolescents and adolescents. Their results revealed a secondary role of interpersonal stressors and pointed to the vital role of self-esteem as a predictor of depression, with negative emotion management being the most important protective factor.

Fifth, Su and Shum tested a model involving critical thinking, cognitive distortions, trait mindfulness and psychological distress on adolescents from Hong Kong. Their results pointed to the important role that mindfulness can have in psychological distress (i.e., depression, anxiety, and stress). At low mindfulness levels, critical thinking was related to greater psychological distress through more cognitive distortions. Given these findings, the authors suggested that mindfulness could have beneficial effects on adolescents' psychological well-being.

Lastly, Kor et al. explored relations between spirituality, character strengths, subjective well-being and prosociality in a longitudinal study of Israeli adolescents. Their findings showed spirituality to be a distinct dimension of character strengths that provided positive development in adolescents and remained stable over time. Furthermore, high levels of spirituality enhanced subjective well-being and prosociality

We hope that the reader will find in this Research Topic a useful reference for the state of the art in the field of well-being of adolescents.

## Author Contributions

LR, MP, and FN: writing, review, and editing.

### Conflict of Interest

The authors declare that the research was conducted in the absence of any commercial or financial relationships that could be construed as a potential conflict of interest.

